# Pursuing healthy homeownership: an evaluation of the neighborhood health trajectories of shared equity homeowners

**DOI:** 10.1186/s12889-024-20982-z

**Published:** 2025-01-02

**Authors:** Geoffrey M. Gusoff, Alex Ramiller, Arthur Acolin, Ruoniu Wang, Frederick J. Zimmerman

**Affiliations:** 1 Department of Family Medicine, David Geffen School of Medicine at UCLA, 10833 Le Conte Ave, Los Angeles, CA 90095 USA; 2 Department of City and Regional Planning, University of California, Berkeley, 230 Bauer Wurster Hall #1820, Berkeley, CA 94720 USA; 3 Runstad Department of Real Estate, 317 Gould Hall, Seattle, WA 98195 USA; 4 Department of Health Policy and Management, Fielding School of Public Health at UCLA, Box 951772, Los Angeles, CA 90095-1772 USA

**Keywords:** Housing and health, Homeownership, Shared equity housing, Community land trusts, Neighborhood health, Walkability, Life expectancy, Food access

## Abstract

**Background:**

Shared equity homeownership – a model in which low- and moderate-income households purchase homes at affordable prices on the condition that the houses remain affordable upon resale – has been shown to produce several health-enhancing housing outcomes. These include permanent affordability, housing stability, and modest wealth-building. However, studies suggest low- and moderate-income households may sacrifice neighborhood quality when becoming homeowners, which can undermine the health benefits of homeownership. To understand the health impacts of the shared equity homeownership model more fully, it is important to evaluate participants’ neighborhood health trajectories – how their neighborhood health environments change when they move into homeownership – and how these trajectories compare to those of similar households entering traditional homeownership and those continuing to rent.

**Methods:**

We conducted difference-in-differences analyses comparing changes in neighborhood health characteristics (walkability, food access, socio-economic vulnerability, and life expectancy) for US households moving into shared equity homeownership between 1997 and 2017 compared to households moving into traditional homeownership and those continuing to rent. Shared equity homeowner data was obtained through the Grounded Solutions Network HomeKeeper National Data Hub and households from the Panel Study of Income Dynamics served as matched controls for the analysis. All data on neighborhood characteristics were obtained from publicly available, census tract-level datasets.

**Results:**

Compared to households entering traditional homeownership, households entering shared equity homeownership experienced a relative increase in walkability (difference-in-differences 1.07, *p* = 0.004), increase in food access (0.13, *p* < 0.001), increase in socio-economic vulnerability (0.06, *p* = 0.02), and similar life expectancy. Compared to households moving between rental units, households entering shared equity homeownership experienced similar trajectories in terms of walkability and food access but experienced a relative increase in socio-economic vulnerability (0.06, *p* = 0.01) and decrease in average neighborhood life expectancy (-0.64, *p* = 0.01).

**Conclusions:**

Households entering shared equity homeownership avoid the sacrifices in neighborhood walkability and food access that are associated with moving into traditional homeownership, but they experience increased neighborhood socio-economic vulnerability. While understanding the net impact of these factors on individual and household health requires further study, these results can inform the siting and design of shared equity homeownership units to maximize the health benefits of the model.

**Supplementary Information:**

The online version contains supplementary material available at 10.1186/s12889-024-20982-z.

## Background

In the search for health-promoting housing policies, homeownership represents a potential double-edged sword. On one hand, homeownership has been associated with various health benefits through the increased stability, housing quality, wealth-building opportunities, and psychological benefits it can provide [1–4]. Given these health benefits, policies that increase homeownership have the potential to significantly improve health outcomes.

On the other hand, there are at least two significant ways in which homeownership may harm health. First, constrained by the locations where they can afford to purchase a home, households may move to neighborhoods with worse place-based determinants of health, including less access to health-related amenities, in the transition from renting to homeownership. One study of low-income, first-time homebuyers in the US found these homebuyers end up in “lower quality” neighborhoods as measured by a variety of socio-economic factors compared to similar households that continue to rent [5]. Another study found that for low- and moderate-income households in the US, becoming a homeowner was associated with living in neighborhoods further from jobs and with higher transportation costs [6]. These findings suggest that for many households, homeownership does require sacrificing important neighborhood characteristics, though the association between homeownership and health-specific neighborhood factors (walkability, food access, etc.) was not evaluated.

Second, the incentives created by traditional homeownership can contribute to broader housing conditions that harm health. To enhance the price of their homes, which is often their most valuable asset, homeowners tend to support restrictive zoning and other drivers of housing scarcity [7]. This not only narrows the scope of people who can obtain the health benefits of homeownership to disproportionately White and well-off households, but may also undermine the affordability of housing for homeowners and renters alike by contributing to overall housing scarcity and price increases [3, 8]. Housing unaffordability and the direct displacement to which it can contribute via foreclosures and evictions are two of the housing-related factors most strongly associated with negative health outcomes [9, 10]. This creates a policy paradox: homeownership has the potential to enhance the health of households who can afford it, but by incentivizing homeowners to maximize their home values by restricting supply, it also has the potential to undermine overall housing affordability, access, and stability, which ultimately undermines health.

### Shared equity homeownership

Shared equity homeownership (SEH) models represent an understudied but promising approach to resolving this policy paradox and improving health through homeownership. Shared equity homeownership models include community land trusts, limited equity cooperatives, and deed-restricted units, all of which use resale restrictions to ensure permanent affordability [11, 12]. In the community land trust model of SEH, a non-profit trust acquires properties, retains ownership of the land, and sells the housing at an affordable price to low- and moderate-income households. In exchange, when the homebuyer sells the house, they are contractually required to sell it at an affordable rate based on a formula determined by the trust [13]. 

To date, there are over 250,000 shared equity housing units across the United States, including over 14,000 homeownership units in community land trusts [14, 15]. SEH models like community land trusts are increasingly capturing the attention of housing advocates and policymakers as they have been shown to provide permanent affordability (typically serving families with incomes 50–120% of area median income), significant stability (one-tenth the rate of foreclosures of traditional homeownership), and modest wealth building ($10,000 per household) as households retain a portion of appreciated value of the home upon resale [11, 16]. In this way, the SEH model includes health-enhancing features of homeownership – stability and modest wealth-building – while improving, rather than undermining, housing affordability and accessibility.

### Assessing neighborhood health trajectories

SEH’s unique combination of affordability, stability, and wealth-building suggest it may provide a healthier alternative to renting or traditional homeownership for low- and moderate-income households. However, to fully consider the extent to which SEH promotes “healthy homeownership”, it is also important to evaluate the neighborhood health environments of SEH participants. Does accessing affordable, stable homeownership through SEH also come with access to neighborhoods with health-enhancing characteristics or does it mean sacrificing healthy neighborhood characteristics like walkability and food access? And how do changes in neighborhood health environments experienced by those moving into SEH compare to changes experienced by similar households that move into traditional homeownership or that continue to rent?

These questions are particularly important for understanding the health impacts of SEH because although SEH programs vary widely in their approaches to identifying and acquiring housing units, SEH properties are sometimes acquired through public land banks that hold vacant or abandoned properties or by organizations aiming to provide homeownership opportunities in historically marginalized neighborhoods [15, 17, 18]. As a result, those moving into SEH may be at particular risk for moving into less healthy neighborhoods. On the other hand, these SEH siting factors might lead SEH units to be concentrated in more urban areas compared to traditional homeownership, potentially providing health-enhancing features like greater walkability [6]. 

To answer these questions, and to more fully understand the health impacts of shared equity homeownership, this study evaluates SEH residents’ “neighborhood health trajectories” – how their neighborhood health environments change when they move into SEH – and how they compare to the neighborhood health trajectories of similar households entering traditional homeownership and to those that continue to rent.

## Methods

### Household data sources & matching

We obtained SEH household data from the Grounded Solutions Network HomeKeeper National Data Hub, a repository of SEH household and transaction information entered by participating SEH organizations [19]. We included all households who purchased or sold an SEH home from 1997 to 2017, who were not previously homeowners before buying an SEH home, and for whom the move origin and destination could be identified at the census tract level. We used the US Census Bureau geocoding tool to match addresses with census tracts. For any unmatched addresses we then used Google’s geocoding tool with manual address corrections to identify the census 2010 tract.

Data for the non-SEH homeowner and renter controls was obtained through the Panel Study of Income Dynamics (PSID) – a longitudinal survey directed by the University of Michigan’s Institute for Social Research [20]. We used restricted-access, bi-annual data that provides 2010 census tract identifiers for household respondents. We included only households that moved between 1997 and 2017, had a head of household at least 18 years old, had an annual income less than $150,000 (approximately the highest income in the HomeKeeper sample), and for whom a pre- and post-move census tract could be identified.

We matched moves recorded in the PSID to HomeKeeper moves, employing a Mahalanobis distance-based matching algorithm from the *matchIt* R package [21]. We performed matching with replacement, so that each PSID household could be matched with multiple HomeKeeper households, allowing for a more balanced comparison between groups. To ensure comparable demographics, starting locational contexts, and life-course characteristics, households were matched based on the following characteristics: householder age, race, and gender, household region and tract density, whether the household included children, whether they moved after 2008, as well as the median home value and median household income for both the household’s region and census tract. Using this approach, we performed 2 distinct matches: (1) households going from renting to shared equity homeownership (“SEH homeowners”) vs. PSID households going from renting to traditional homeownership (“PSID homeowners”) and (2) households going from renting to shared equity homeownership vs. PSID households going from renting to renting (“PSID renters”).

### Outcome data sources & measures

To evaluate features of neighborhood health environments, we used publicly available census tract-level data on the built environment, focusing on walkability and food access, as well as an index of socioeconomic vulnerability as a measure of neighborhood-level socioeconomic determinants of health. These neighborhood features were chosen given their association to individual- and community-level health outcomes [22–24]. We also included data on average life expectancy for people residing in the neighborhood as a proxy for the cumulative impact of neighborhood environmental factors [25]. 

To measure walkability we used the Environmental Protection Agency’s National Walkability Index, a composite index of built environment features influencing the likelihood of people walking as a mode of transportation, including proximity to transit, density of street intersections, and land use diversity [26]. The data comes primarily from census-related measures collected between 2014 and 2020 [27]. The index ranges from 1 to 20, representing ranked quantiles such that tracts with a score of 1 are in the bottom 5% of tracts in terms of walkability features and those with a score of 20 are in the top 5%.

To measure food access we used the 2010 version of the U.S. Department of Agriculture’s Food Access Research Atlas (FARA). The Atlas provides a binary variable for each census tract stating whether it is low-access (whether at least 500 people or 33% of the population live greater than 0.5 miles from the nearest grocery store or supermarket for urban areas or more than 10 miles for rural areas) [28]. 

To measure neighborhood socioeconomic characteristics, we used the socioeconomic domain of the 2010 version of the Center for Disease Control’s Social Vulnerability Index (SVI). The SVI ranks the social vulnerability of all census tracts by their percentile (ranging from 0 to 1 with 1 reflecting the highest vulnerability) overall and across particular domains [29]. Our analysis focused on the socioeconomic domain of SVI (SVI-SES) given the strong links between the socioeconomic factors measured (income, employment, educational attainment) and local health outcomes [30]. All estimates are derived from the 2006–2010 American Community Survey.

Finally, to measure life expectancy we used the Center for Disease Control’s U.S. Small-Area Life Expectancy Estimates Project (USALEEP) [31]. USALEEP provides life expectancy at birth estimates for each census tract based on vital statistics collected between 2010 and 2015.

For data sources that provided data from multiple years, FARA and SVI-SES, we used the version for which data collection occurred near or during the weighted center of our HomeKeeper data (approximately 2010).

### Statistical analysis

To account for baseline differences in neighborhood characteristics between the shared equity homeowners and control groups and to isolate the effects of moving into SEH, we conducted a weighted linear difference-in-differences analysis for each variable:

$$Outcom{e_i}\, = \,SE{H_i}\, + \,Perio{d_i}\, + \,SE{H_i}*\,Perio{d_i}\, + \,{e_{it}}$$

For each of the two matched datasets (SEH homeowners matched to PSID homeowners and SEH homeowners matched to PSID renters), we ran a separate analysis on each of the four outcome variables (walkability, food access, socioeconomic vulnerability, and life expectancy). *SEH* is a binary variable that equals 1 for HomeKeeper data records. *Period* is a binary variable where the reference period is the time before moving into SEH and the later period is the period living in SEH. SEH * Period, the interaction between SEH exposure and time period, is the primary observation of interest and indicates if there is a statistically significant difference in the change of neighborhood characteristics before and after moves between the PSID and SEH groups. We used a weighting approach (multiplying matching weights by PSID sampling weights for PSID observations) to promote optimal matching balance and account for the PSID sampling structure.

We conducted a sensitivity analysis excluding the Champlain Housing Trust in Vermont – the largest shared equity homeownership organization in the dataset representing 18% of all SEH observations – to assess the extent to which unique features of the Vermont context (e.g. relatively high life expectancy, relatively low racial/ethnic diversity) influenced the results of the overall analysis. In addition, we conducted a supplementary difference-in-differences analysis comparing both control groups to each other (PSID homeowners vs. PSID renters) to more directly assess the differences in neighborhood health trajectories between households moving into traditional homeownership and those that continue to rent.

Finally, to assess for the stability of neighborhood health measures across years, we calculated the intraclass correlation (ICC) one-way random effects model for census tracts across each dataset year for the FARA and SVI-SES. The ICC model quantifies the variation of the observations over the different time periods, with values of 0.8 or above suggestive of relatively high consistency over time [32]. 

## Results

After applying the inclusion criteria to the HomeKeeper database and matching participants’ census tracts to data across all four neighborhood health datasets, we obtained data for 3,858 SEH homeowners. Table 1 presents the weighted baseline (pre-move) characteristics of SEH homeowners and the PSID comparison groups as well as the standardized mean distance (SMD) between the groups. The SMD provides a measure of the degree of balance for each of the matching variables between the control and treatment groups. [33, 34] We calculate the SMD as the difference in means between the treatment and control groups, divided by the standard deviation of the treatment group prior to the match. [34] Overall, the baseline characteristics are similar between groups. However, SEH homeowners were less likely to be White, more likely to have children and more likely to have a female head of household than the matched PSID respondents. In terms of pre-move neighborhood health characteristics, SEH homeowners had a somewhat higher walkability score, lower food access, and lower socioeconomic vulnerability than the PSID groups.

**Table 1:** Weighted Baseline (Pre-Move) Characteristics and Standardized Mean Differences (SMD) between SEH and PSID Groups

Variable (weighted)	SEH (*n* = 3858)	PSID Rent-Own (*n* = 927)	**SMD**	PSID Rent-Rent (*n* = 1726)	**SMD**
**Matching Variables**					
Householder Age, years	38.0	39.4	-0.12	39.8	-0.14
Householder Race/Ethnicity					
Black, %	12.7	8.0	0.05	13.1	-0.0
Latinx, %	12.4	9.7	0.03	12.7	-0.0
White, %	61.6	76.6	-0.15	71.3	-0.1
Female-Headed Household, %	61.8	54.4	0.07	55.0	0.07
Children in Household, %	51.9	36.5	0.15	36.8	0.15
Household Income, mean	$44,374	$48,949	-0.19	$44,101	0.01
Median Household Income (Region)	$57,524	$57,800	-0.11	$57,498	0.01
Median Home Value (Region)	$380,790	$372,594	0.08	$376,816	0.04
Median Household Income (Tract)	$63,089	$63,077	0.01	$63,014	0.04
Median Home Value (Tract)	$248,504	$244,114	0.24	$246,347	0.12
Population Density	5,716	4,332	0.15	5,105	0.07
**Outcome Variables**					
Walkability Score	12.4	10.6		11.2	
Food Access, %	76.2	82.0		77.5	
SVI-SES Ranking	0.43	0.49		0.52	
Life Expectancy, mean	79.4	78.6		78.3	

**Fig. 1:**
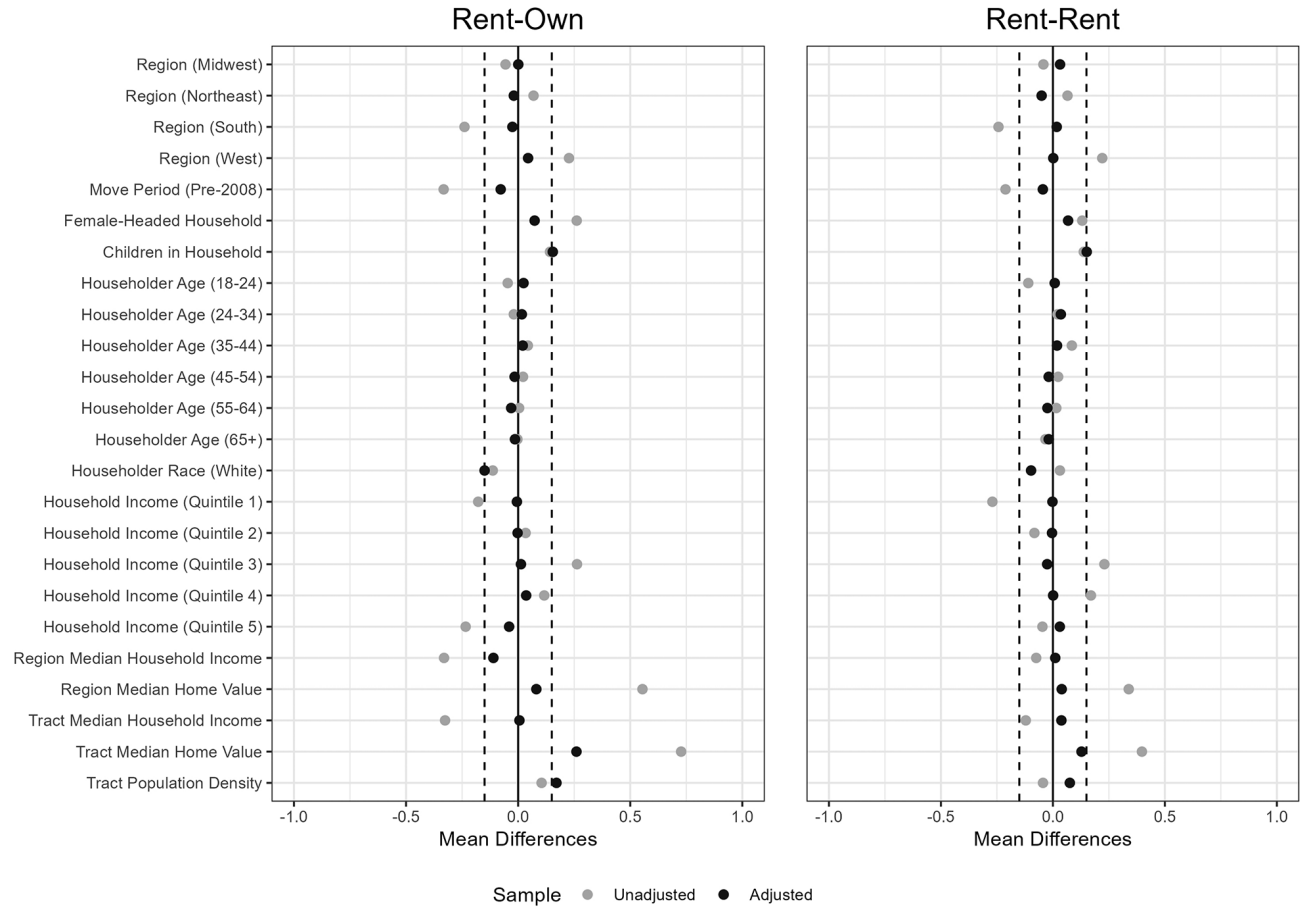
Match balance improvements for variables in each matching model, measured by standardized mean difference

The match balance for each of the comparisons across the matched characteristics is presented in Fig. 1. Overall, the matching strategy is effective at matching the variables of interest. Standard thresholds used in the literature generally consider an absolute SMD of less than 0.1 as a good match, indicating that the mean difference between the treatment and control groups is one tenth the size of the standard deviation, and 0.3 as an acceptable match. [33, 34] Most matching variables have a Standardized Mean Distance (SMD) between − 0.1 and 0.1 and all of the variables have an absolute SMD less than 0.3. 

**Fig. 2:**
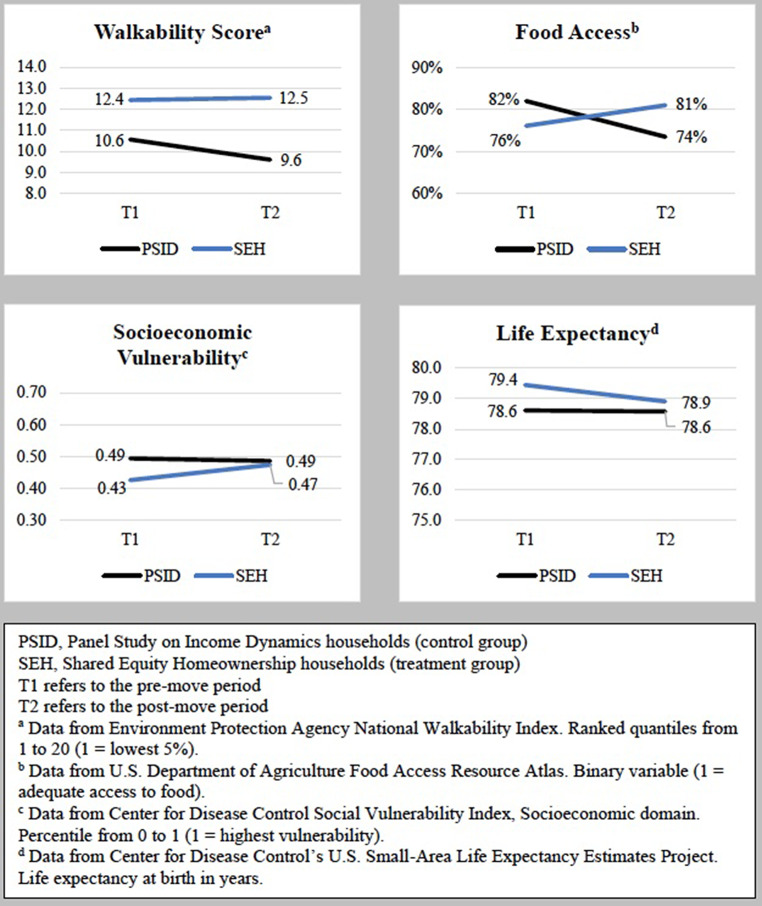
Graphs of difference-in-differences results of households entering SEH versus PSID households entering traditional homeownership

Table 2; Fig. 2 present the results of the difference-in-differences analyses comparing changes in neighborhood health characteristics for SEH homeowners versus PSID homeowners. For PSID homeowners, moving to homeownership was associated with a significant decrease in the walkability score (from 10.6 to 9.6). In contrast, the walkability score for SEH homeowners remained relatively stable pre- and post-move, yielding an overall walkability score difference in differences of 1.07 (*p* < 0.01). Moving to homeownership was also associated with a decrease in the proportion of PSID homeowners in neighborhoods with adequate food access (from 82 to 74%). In contrast, moving to homeownership was associated with an increase in the proportion of SEH homeowners in neighborhoods with adequate food access (from 76 to 81%), with an overall difference in differences of 13% (*p* < 0.001) between SEH and PSID homeowners pre- to post-move.

SEH homeowners had lower neighborhood socioeconomic vulnerability indices than PSID homeowners in the pre-move period (0.43 vs. 0.49). During their moves into homeownership, neighborhood SVI-SES increased for SEH homeowners while neighborhood SVI-SES remained stable for PSID homeowners, for an overall SVI-SES difference in differences of 0.06 (*p* = 0.02). Finally, average neighborhood life expectancy was higher among SEH homeowners than PSID homeowners pre-move (79.4 years vs. 78.6 years), but there was no significant move-related difference in differences in neighborhood life expectancy across the two groups.

**Table 2:** Results of difference-in-differences analysis of households entering SEH versus PSID households entering traditional homeownership

	Walkability Score^a^	Food Access^b^	SVI-SES^c^	Life Expectancy^d^
Intercept (SD) p-value	10.6 (0.23) *< 0.001*	0.82 (0.02) *< 0.001*	0.49 (0.02) *< 0.001*	78.6 (0.24) *< 0.001*
SEH (SD) p-value	1.88 (0.24) *< 0.001*	–0.06 (0.02) *0.01*	–0.07 (0.02) *< 0.001*	0.84 (0.25) *0.001*
Period (SD) p-value	–0.96 (0.36) *0.01*	–0.09 (0.04) *0.03*	–0.01 (0.02) *0.73*	–0.03 (0.32) *0.92*
SEH * Period (SD) *p-value*	1.07 (0.37) *0.004*	0.13 (0.04) *0.001*	0.06 (0.02) *0.02*	–0.51 (0.33) *0.12*

Treatment Observations 3,858

Control Observations 927

SD, standard deviation

^a^ Data from Environment Protection Agency National Walkability Index. Ranked quantiles from 1 to 20 (1 = lowest 5%)

^b^ Data from U.S. Department of Agriculture Food Access Resource Atlas. Binary variable (1 = adequate access to food)

^c^ Data from Center for Disease Control Social Vulnerability Index, Socioeconomic domain. Percentile from 0 to 1 (1 = highest vulnerability)

^d^ Data from Center for Disease Control’s U.S. Small-Area Life Expectancy Estimates Project. Life expectancy at birth in years

**Fig. 3:**
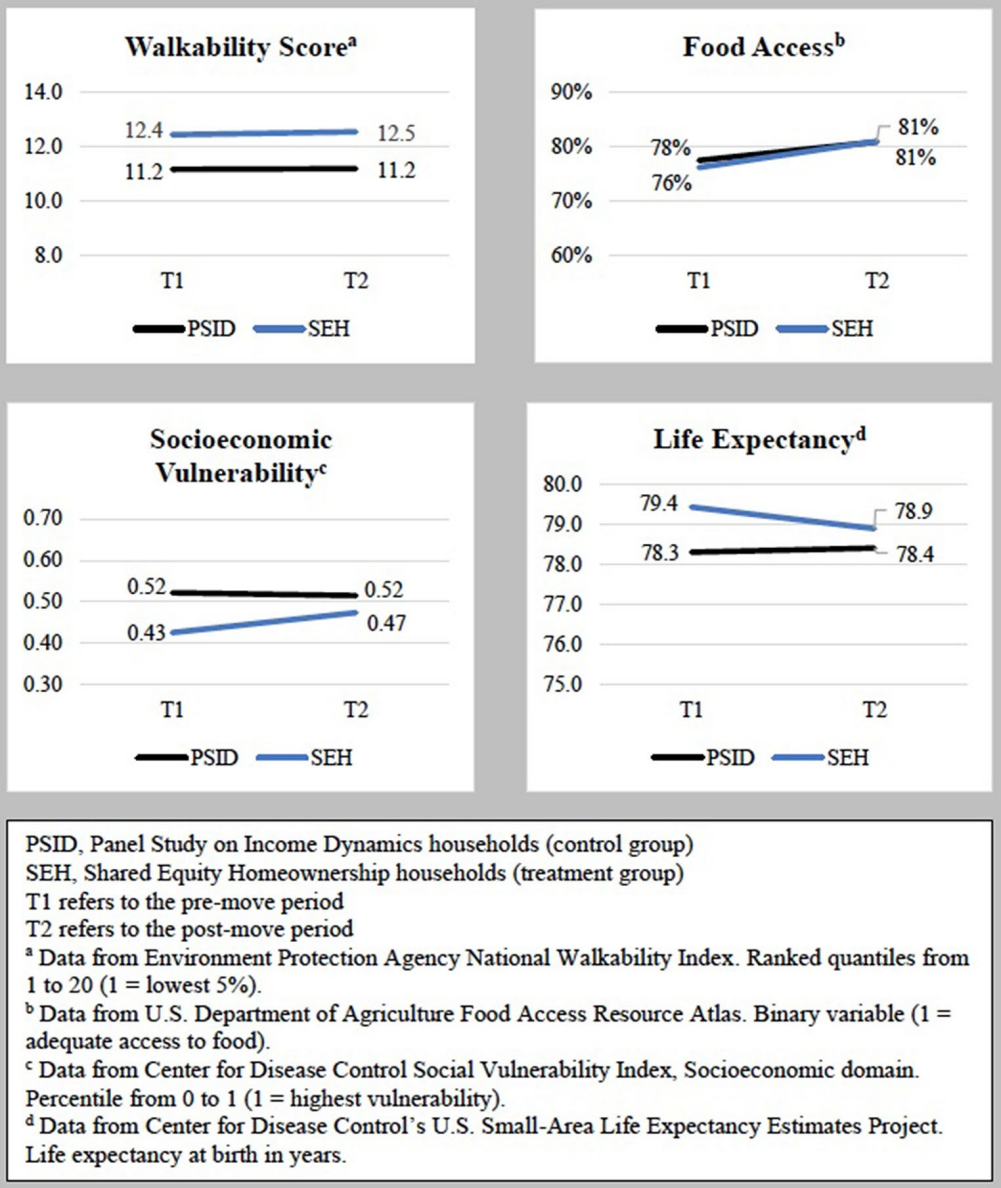
Graphs of difference-in-differences results of households entering SEH versus PSID households continuing to rent

Table 3; Fig. 3 present the results of the difference-in-differences analyses comparing changes in neighborhood health characteristics for SEH homeowners versus comparable PSID renters. The pre-move walkability score was higher among SEH homeowners than PSID renters (12.4 vs. 11.2), but there was no significant difference in differences in walkability score associated with moving. The percentage of SEH homeowners and PSID renters living in neighborhoods with adequate food access was similar in the pre-move period, and there was no significant difference-in-differences in neighborhood food access associated with moving.

Compared to PSID renters, SEH homeowners lived in neighborhoods with lower SVI-SES in the pre-move period (0.52 vs. 0.43). Moves were associated with a larger increase in neighborhood SVI-SES for SEH households than for PSID renters with a difference-in-differences of 0.06 (*p* = 0.01). Finally, pre-move neighborhood life expectancy was higher among SEH homeowners than PSID renters (79.4 years vs. 78.3 years). Moves were associated with a decrease in neighborhood life expectancy for SEH homeowners but not for PSID renters, yielding a pre/post move difference-in-differences in neighborhood life expectancy of -0.64 years (*p* = 0.01) between the groups.

**Table 3:** Results of difference-in-differences analysis of households entering SEH versus PSID households continuing to rent

	Walkability Score^a^	Food Access^b^	SVI-SES^c^	Life Expectancy^d^
Intercept (SD) p-value	11.2 (0.16) *< 0.001*	0.78 (0.02) *< 0.001*	0.52 (0.01) *< 0.001*	78.3 (0.18) *< 0.001*
SEH (SD) p-value	1.27 (0.17) *< 0.001*	–0.01 (0.02) *0.51*	–0.10 (0.01) *< 0.001*	1.13 (0.19) *< 0.001*
Period (SD) p-value	0.03 (0.22) *0.91*	0.03 (0.03) *0.18*	-0.01 (0.02) *0.74*	0.10 (0.24) *0.67*
SEH * Period (SD) p-value	0.08 (0.23) *0.72*	0.01 (0.03) *0.60*	0.06 (0.02) *0.01*	–0.64 (0.26) *0.01*

Treatment Observations 3,858

Control Observations 1,726

SD, standard deviation

^a^ Data from Environment Protection Agency National Walkability Index. Ranked quantiles from 1 to 20 (1 = lowest 5%)

^b^ Data from U.S. Department of Agriculture Food Access Resource Atlas. Binary variable (1 = adequate access to food)

^c^ Data from Center for Disease Control Social Vulnerability Index, Socioeconomic domain. Percentile from 0 to 1 (1 = highest vulnerability)

^d^ Data from Center for Disease Control’s U.S. Small-Area Life Expectancy Estimates Project. Life expectancy at birth in years

In the sensitivity analysis excluding the Champlain Housing Trust in Vermont, the largest SEH site in the dataset, the difference-in-differences were of similar magnitude, direction, and statistical significance as in the main model (Additional Files S1 and S2).

The difference-in-differences analysis comparing PSID homeowners and PSID renters showed similar trajectories for each group as in the analyses comparing them to SEH homeowners (Additional File S3). Compared to PSID renters, PSID homeowners experienced relative decreases in walkability (-0.98, *p* = 0.02) and food access (-12%, *p* < 0.01), while changes in neighborhood socioeconomic vulnerability and life expectancy were similar between both groups.

The ICC one-way random effects model revealed high levels of consistency in neighborhood characteristics across years for all measures that were collected at multiple times (ICC = 0.89 for food access and 0.92 for SVI-SES, respectively) (Additional File S4).

## Discussion

We found that for low- and moderate-income households, moving into traditional homeownership was associated with a decline in neighborhood walkability and food access but no significant change in neighborhood socioeconomic vulnerability or life expectancy. In contrast, for low- and moderate-income households moving into SEH, moving was associated with improved food access, increases in neighborhood socioeconomic vulnerability, and relatively stable levels of neighborhood walkability and life expectancy. For low- and moderate-income households who remained renters, moving was not associated with any significant change in neighborhood walkability, food access, socioeconomic vulnerability, or life expectancy.

These results suggest low- and moderate-income households on average sacrifice some level of neighborhood walkability and food access when moving to traditional homeownership. Moving into SEH avoids these sacrifices and even increases the chances of living in a neighborhood with sufficient food access. This is consistent with findings from interviews with SEH residents and staff, who identified walkability and greater access to local stores as important features of SEH [35]. 

These findings also have important health implications. Higher neighborhood walkability has been associated with decreased risk for developing diabetes and cardiovascular disease [22, 36]. The presence of local grocery stores has also been linked to improved health outcomes [23, 37]. Of note, the supply of local grocery stores alone does not automatically lead to an increase in healthy food consumption among low-income households, which also requires addressing “demand” factors like households’ disposable income [38]. However, by ensuring housing affordability, the SEH model also has the potential to improve households’ disposable income so they can better access healthy foods at local grocery stores [39]. 

The results also suggest that moving into SEH generally means moving into a neighborhood with higher socioeconomic vulnerability, a difference not seen in either of the PSID control groups. Higher overall neighborhood social vulnerability (which also takes into account demographics regarding age, race, and primary language as well as housing stock characteristics) has been associated with worse health outcomes, such as higher rates of chronic disease and worse surgical outcomes [40, 41]. In addition, moving into neighborhoods with increased socioeconomic vulnerability, especially concentrated poverty, has been associated with worse physical and mental health outcomes [24, 42]. However, these studies generally assessed larger differences in neighborhood socioeconomic vulnerability, and further research is needed to determine the extent to which smaller changes in the middle of the socioeconomic vulnerability range impact the health of households.

Finally, we found neighborhood life expectancy decreased 0.64 years for households moving into SEH relative to PSID households that remained renters. Life expectancy is an important measure of the health status of current residents and partially reflects neighborhood exposures (e.g. toxins, the built environment, socioeconomic conditions, violence, etc.) that could impact the health of residents moving into those neighborhoods [25]. At the same time, life expectancy in a neighborhood may also reflect more remote exposures previously experienced by current residents (e.g. lack of green spaces that has since improved) that may not impact people moving into those neighborhoods. In this way, further research of specific neighborhood exposures is needed to determine the extent to which the neighborhood life expectancy decline associated with moving into SEH reflects a health risk to households moving into SEH.

Overall, moving into SEH appears to avoid the decline in walkability and food access that low- and moderate-income households generally face when moving into traditional homeownership. Moving into SEH is also associated with modest increases in neighborhood socioeconomic vulnerability relative to households moving into traditional homeownership and those that continue to rent and modest decreases in neighborhood life expectancy relative to households remaining renters, which may reflect worsening of some neighborhood health exposures.

### Limitations

This study has several limitations that should be addressed in future studies. First, the neighborhood health factors assessed provide an important but incomplete picture of local environmental exposures. Future studies should incorporate additional tract-level data on the social, economic, and built environment (e.g. pollutants, green spaces, healthcare facility access, etc.) as these data become increasingly available to provide a fuller picture of neighborhood health environments.

Second, the neighborhood health data was sometimes collected years before or after the observed moves took place. This could potentially limit the accuracy of findings in neighborhoods that underwent significant changes in the interim, though our analysis of the multi-year datasets suggests relative stability in those measures over time (Additional File S4).

Third, the study assesses moves over a twenty-year period during which local housing markets and households’ moving behaviors may have changed significantly, particularly in the aftermath of the Great Recession and foreclosure crisis. Our analysis matched groups by whether a move occurred after 2008 to account for time-based variation related to the foreclosure crisis. Given our sample size, subperiod analysis was not possible. As the number of SEH observations continues to grow, future studies should match on more granular time variables enabled by larger sample sizes to more fully account for potential time-based variation in household trajectories.

Fourth, while the matching strategy was effective at matching SEH and PSID control groups on measured characteristics, the groups may differ on unmeasured characteristics. Additional data on salient household characteristics, such as a households’ motivation for moving (e.g. forced displacement vs. voluntary moves) and neighborhood preferences, could provide even more robust matching in future analyses. In addition, qualitative studies of households entering SEH versus traditional homeownership could provide a fuller picture of the motivations and preferences driving household moving decisions and the trade-offs households face when pursuing different housing options.

Finally, the distribution of SEH homeownership units in the dataset, which includes significant representation from larger and more established SEH communities, may limit the extent to which the findings can be generalized to all SEH communities. However, many smaller and newer SEH communities were included in the sample and the sensitivity analysis, which excluded the largest SEH community in Vermont, were reassuring against the concern that a specific large community might be driving the main findings. As the SEH model continues to grow across the country, it will be important to include SEH households from diverse settings in future analyses to further increase the generalizability of findings.

### Policy Implications

The stability, affordability, and modest wealth-building SEH provides for low- and moderate-income households have important health benefits for those households. Our findings suggest that moving into SEH is also associated with positive neighborhood health exposures like walkable environments and greater food access, but increased socioeconomic vulnerability and decreased life expectancy may reflect some negative neighborhood health exposures as well.

With the skyrocketing costs of housing, there is a risk that future SEH will be concentrated in lower-cost, less-resourced neighborhoods to reduce the costs of development. To maximize the health benefits of SEH, it will be important for public entities and the non-profits who develop SEH units to find creative ways to site SEH in healthy environments. This may require incorporating neighborhood health environment measures as explicit criteria for site selection as well as deploying strategies such as re-zoning, converting public space for SEH development, increasing public investment in under-resourced communities, and developing new amenities like green spaces or grocery stores in SEH neighborhoods.

## Conclusion

For low- and moderate-income households, moving into SEH represents a way to participate in the benefits of homeownership without sacrificing neighborhood walkability and food access, but it also involves moving into a neighborhood with higher socioeconomic vulnerability. Determining the overall health impact of SEH through both household-level factors (stability, affordability, wealth-building) and neighborhood-level factors requires further study. Public entities and non-profit SEH developers can improve the latter through health-conscious siting of SEH units and the development of healthy amenities within SEH communities.

## Electronic Supplementary Material

Below is the link to the electronic supplementary material.

**Additional File 1** Results of difference-in-differences analysis of households entering SEH versus PSID households entering traditional homeownership excluding Champlain Housing Trust Description of Data: Table displaying the difference-in-difference results comparing neighborhood health trajectories for households entering shared equity homeownership versus those of PSID households entering traditional homeownership, with the largest shared equity homeownership entity (Champlain Housing Trust) excluded from the analysis.

**Additional File 2** Results of difference-in-differences analysis of households entering SEH versus PSID households continuing to rent excluding Champlain Housing Trust Description of Data: Table displaying the difference-in-difference results comparing neighborhood health trajectories for households entering shared equity homeownership versus those of PSID households continuing to rent, with the largest shared equity homeownership entity (Champlain Housing Trust) excluded from the analysis.

**Additional File 3** Results of difference-in-differences analysis of PSID households entering traditional homeownership versus PSID households continuing to rent Description of Data: Table displaying the difference-in-difference results comparing neighborhood health trajectories for PSID households entering traditional homeownership versus those of PSID households continuing to rent.

**Additional File 4** Assessment of variable stability over time. Description of Data: Table displaying the intraclass correlation (ICC) between various waves of data for the study variables for which multiple years of data were available (food access and the socioeconomic domain of the social vulnerability index).

## Data Availability

Data that support the findings of this study are available from Grounded Solutions Network and the Panel Study of Income Dynamics but restrictions apply to the availability of these data, which were used under data sharing agreements for the current study, and so are not publicly available. Code developed for the analysis is however available from the authors upon reasonable request and data can be obtained with the permission of Grounded Solutions Network and the Panel Study on Income Dynamics.

## Abbreviations

FARA: Food Access Research Atlas
ICC: Intraclass correlation
PSID: Panel Study of Income Dynamics
SD: Standard Deviation
SEH: Shared Equity Homeownership
SMD: Standardized Mean Distance
SVI: Social Vulnerability Index
SVI: SES–Social Vulnerability Index–Socioeconomic Domain
USALEEP: U.S. Small–Area Life Expectancy Estimates Project

## References

[1] Dietz RD, Haurin DR. The social and private micro-level consequences of homeownership. J Urban Econ. 2003;54(3):401–50.

[2] Lindblad MR, Manturuk KR, Quercia RG. Sense of Community and Informal Social Control among Lower Income households: the role of homeownership and collective efficacy in reducing subjective Neighborhood Crime and Disorder. Am J Community Psychol. 2013;51(1–2):123–39. 22484395 10.1007/s10464-012-9507-9

[3] Gusoff G, Chen K, Moreno G, Elmore JG, Zimmerman FJ. The Relationship between Homeownership and Health by Race/Ethnicity since the Foreclosure Crisis: California Health interview Survey 2011–2018. J Gen Intern Med. 2023;38(12):2718–25. 37227660 10.1007/s11606-023-08228-x PMC10506978

[4] Boehm TP, Schlottmann A. Wealth Accumulation and Homeownership: evidence for low-income households. Cityscape. 2008;10(2):225–56.

[5] Van Zandt S, Rohe WM. Do first-time home buyers improve their Neighborhood Quality? J Urban Affairs. 2006;28(5):491–510.

[6] Ramiller A, Acolin A, Walter RJ, Wang R. Moving to shared equity: locational outcomes for households in shared equity homeownership programs. Hous Stud. 2022;39(5):1239–63.10.1080/02673037.2022.2115467 10.1080/02673037.2022.2115467 PMC11213554 38948154

[7] Fischel WA. The homevoter hypothesis: how home values influence local government taxation, school finance, and land-use policies. Harvard University Press Cambridge, MA; 2002.

[8] Hankinson M. When do renters behave like homeowners? High Rent, price anxiety, and NIMBYism. Am Polit Sci Rev. 2018;112(3):473–93.

[9] Taylor L. Housing and health: an overview of the literature. Health Affairs; 2018.

[10] Downing J. The health effects of the foreclosure crisis and unaffordable housing: a systematic review and explanation of evidence. Soc Sci Med. 2016;162:88–96. 27343818 10.1016/j.socscimed.2016.06.014

[11] Acolin A, Ramiller A, Walter RJ, Thompson S, Wang R. Transitioning to Homeownership: Asset Building for low-and moderate-income households. Hous Policy Debate. 2021;31(6):1032–49. 34866882 10.1080/10511482.2021.1949372 PMC8635301

[12] Thaden E, Greer A, Saegert S. Shared Equity Homeownership: a welcomed Tenure Alternative among Lower Income households. Hous Stud. 2013;28(8):1175–96.

[13] Hindman DJ, Pollack CE. Community Land Trusts as a Means to improve Health. JAMA Health Forum. 2020;1(2):e200149. 36218645 10.1001/jamahealthforum.2020.0149

[14] Thaden E. The State of Shared-Equity Homeownership. Shelterforce. 2018.

[15] Wang RV, Wandio C, Bennett A, Spicer J, Corugedo S, Thaden E. The 2022 Census of Community Land Trusts and Shared Equity Entities in the United States: Prevalence, Practice and Impact. 2023.

[16] Thaden E. Stable home ownership in a turbulent economy: delinquencies and foreclosures remain low in community land trusts. Lincoln Institute of Land Policy.; 2011.

[17] Lowe JS, Prochaska N, Keating WD. Bringing permanent affordable housing and community control to scale: the potential of community land trust and land bank collaboration. Cities. 2022;126:103718.

[18] Fujii Y. Putting the pieces together: how collaboration between land banks and community land trusts can promote affordable housing in distressed neighborhoods. Cities. 2016;56:1–8.

[19] Wang R, Cahen C, Acolin A, Walter RJ. Tracking growth and evaluating performance of shared equity homeownership programs during housing market fluctuations. JSTOR; 2019.

[20] Johnson D, McGonagle K, Freedman V, Sastry N. Fifty years of the Panel Study of Income dynamics: Past, Present, and Future. Ann Am Acad Pol Soc Sci. 2018;680(1):9–28. 31666744 10.1177/0002716218809363 PMC6820672

[21] Stuart EA, King G, Imai K, Ho D. MatchIt: nonparametric preprocessing for parametric causal inference. J Stat Softw. 2011;42(8). https://www.jstatsoft.org/article/view/v042i08

[22] Dendup T, Feng X, Clingan S, Astell-Burt T. Environmental risk factors for developing type 2 diabetes Mellitus: a systematic review. Int J Environ Res Public Health. 2018;15(1). 10.3390/ijerph15010078 PMC5800177 29304014

[23] Moughames E, Woo H, Galiatsatos P, Romero-Rivero K, Raju S, Tejwani V, et al. Disparities in access to food and chronic obstructive pulmonary disease (COPD)-related outcomes: a cross-sectional analysis. BMC Pulm Med. 2021;21(1):139. 33906617 10.1186/s12890-021-01485-8 PMC8077917

[24] Ludwig J, Sanbonmatsu L, Gennetian L, Adam E, Duncan GJ, Katz LF, et al. Neighborhoods, obesity, and diabetes–a randomized social experiment. N Engl J Med. 2011;365(16):1509–19. 22010917 10.1056/NEJMsa1103216 PMC3410541

[25] Life expectancy by. County, race, and ethnicity in the USA, 2000-19: a systematic analysis of health disparities. Lancet. 2022;400(10345):25–38. 35717994 10.1016/S0140-6736(22)00876-5 PMC9256789

[26] Thomas J, Reyes R. National walkability index. Methodology and user guide. United States Environmental Protection Agency (EPA) https://www.epa.gov/sites/default/files/2021-06/documents/national_walkability_index_methodology_and_user_guide_june2021.pdf 2021.

[27] Chapman J, Fox EH, Bachman W, Frank LD, Thomas J, Reyes AR. Smart location database technical documentation and user guide. Washington, DC: US Environmental Protection Agency; 2021.

[28] Economic Research Service USDoA. Food Access Research Atlas: Documentation Washington, D.C.2022. https://www.ers.usda.gov/data-products/food-access-research-atlas/documentation/

[29] Flanagan BE, Hallisey EJ, Adams E, Lavery A. Measuring Community vulnerability to natural and anthropogenic hazards: the Centers for Disease Control and Prevention’s Social Vulnerability Index. J Environ Health. 2018;80(10):34–6. 32327766 PMC7179070

[30] Dwyer-Lindgren L, Bertozzi-Villa A, Stubbs RW, Morozoff C, Mackenbach JP, van Lenthe FJ, et al. Inequalities in Life Expectancy among US counties, 1980 to 2014: temporal trends and Key drivers. JAMA Intern Med. 2017;177(7):1003–11. 28492829 10.1001/jamainternmed.2017.0918 PMC5543324

[31] Arias E, Escobedo LA, Kennedy J, Fu C, Cisewski JA. US small-area life expectancy estimates project: Methodology and results summary. 2018. 30312153

[32] Liljequist D, Elfving B, Skavberg Roaldsen K. Intraclass correlation - A discussion and demonstration of basic features. PLoS ONE. 2019;14(7):e0219854. 31329615 10.1371/journal.pone.0219854 PMC6645485

[33] Austin PC. Balance diagnostics for comparing the distribution of baseline covariates between treatment groups in propensity-score matched samples. Stat Med. 2009;28(25):3083–107. 19757444 10.1002/sim.3697 PMC3472075

[34] Zhang Z, Kim HJ, Lonjon G, Zhu Y. Balance diagnostics after propensity score matching. Ann Transl Med. 2019;7(1):16. 30788363 10.21037/atm.2018.12.10 PMC6351359

[35] Rose J, Arikat L, Gusoff G, Pollack CE. Mechanisms to Improve Health through Community Land trusts. J Urban Health. 2023;100(2). https://link.springer.com/article/10.1007/s11524-022-00706-7 10.1007/s11524-022-00706-7 PMC9869833 36689141

[36] Makhlouf MHE, Motairek I, Chen Z, Nasir K, Deo SV, Rajagopalan S, et al. Neighborhood Walkability and Cardiovascular Risk in the United States. Curr Probl Cardiol. 2023;48(3):101533. 36481391 10.1016/j.cpcardiol.2022.101533 PMC9892210

[37] Tipton MJ, Wagner SA, Dixon A, Westbay L, Darji H, Graziano S. Association of Living in a Food Desert with pregnancy morbidity. Obstet Gynecol. 2020;136(1):140–5. 32541293 10.1097/AOG.0000000000003868

[38] Allcott H, Diamond R, Dubé J-P, Handbury J, Rahkovsky I, Schnell M. Food deserts and the causes of nutritional inequality. Q J Econ. 2019;134(4):1793–844.

[39] Kirkpatrick SI, Tarasuk V. Housing circumstances are associated with household food access among low-income urban families. J Urban Health. 2011;88(2):284–96. 21286826 10.1007/s11524-010-9535-4 PMC3079041

[40] Jain V, Al Rifai M, Khan SU, Kalra A, Rodriguez F, Samad Z, et al. Association between social vulnerability index and cardiovascular disease: a behavioral risk factor surveillance system study. J Am Heart Assoc. 2022;11(15):e024414. 35904206 10.1161/JAHA.121.024414 PMC9375494

[41] Paro A, Hyer JM, Diaz A, Tsilimigras DI, Pawlik TM. Profiles in social vulnerability: the association of social determinants of health with postoperative surgical outcomes. Surgery. 2021;170(6):1777–84. 34183179 10.1016/j.surg.2021.06.001

[42] Gennetian LA, Ludwig J, McDade T, Sanbonmatsu L. Why concentrated poverty matters. Pathways Spring 2013. 2013:10–3. https://cpi.stanford.edu/_media/pdf/pathways/spring_2013/Pathways_Spring_2013_Gennetian_Ludwig_McDade_Sanbonmatsu.pdf

