# Supplementary material for: Pursuing healthy homeownership: an evaluation of the neighborhood health trajectories of shared equity homeowners
Source: BMC Public Health. 2025 Jan 2;25:11. doi: 10.1186/s12889-024-20982-z (PMC11697963; doi:10.1186/s12889-024-20982-z)
Supplement: Supplementary file 2 — Additional File 2 Results of difference-in-differences analysis of households entering SEH versus PSID households continuing to rent excluding Champlain Housing Trust Description of Data: Table displaying the difference-in-difference results comparing neighborhood health trajectories for households entering shared equity homeownership versus those of PSID households continuing to rent, with the largest shared equity homeownership entity (Champlain Housing Trust) excluded from the analysis. [file 12889_2024_20982_MOESM2_ESM.docx]

**Additional File 2. Results of difference-in-differences analysis of households entering SEH versus PSID households continuing to rent excluding the Champlain Housing Trust**

|  | **Walkability Score^a^** | **Food Access^b^** | **SVI (SES)^c^** | **Life Expectancy^d^** |
| --- | --- | --- | --- | --- |
| Intercept  (SD)  *p-value* | 11.36  (0.18)  *<0.001* | 0.79  (0.02)  *<0.001* | 0.53  (0.02)  *<0.001* | 78.34  (0.19)  *<0.001* |
| SEH  (SD)  *p-value* | 1.23  (0.19)  *<0.001* | –0.02  (0.02)  *0.28* | –0.09  (0.02)  *<0.001* | 0.90  (0.20)  *<0.001* |
| Period  (SD)  *p-value* | –0.02  (0.24)  *0.93* | 0.03  (0.03)  *0.23* | -0.00  (0.02)  *0.86* | 0.02  (0.27)  *0.94* |
| SEH * Period  (SD)  *p-value* | 0.17  (0.25)  *0.50* | 0.02  (0.03)  *0.41* | 0.07  (0.02)  *0.004* | –0.67  (0.29)  *0.02* |
| Treatment Observations 3,161  Control Observations 1,471 | | | | |
| SD, standard deviation  ^a^ Data from Environment Protection Agency National Walkability Index. Ranked quantiles from 1 to 20 (1 = lowest 5%).  ^b^ Data from U.S. Department of Agriculture Food Access Resource Atlas. Binary variable (1 = adequate access to food).  ^c^ Data from Center for Disease Control Social Vulnerability Index, Socioeconomic domain. Percentile from 0 to 1 (1 = highest vulnerability).  ^d^ Data from Center for Disease Control’s U.S. Small-Area Life Expectancy Estimates Project. Life expectancy at birth in years. | | | | |
