# Supplementary material for: Pursuing healthy homeownership: an evaluation of the neighborhood health trajectories of shared equity homeowners
Source: BMC Public Health. 2025 Jan 2;25:11. doi: 10.1186/s12889-024-20982-z (PMC11697963; doi:10.1186/s12889-024-20982-z)
Supplement: Supplementary file 3 — Additional File 3 Results of difference-in-differences analysis of PSID households entering traditional homeownership versus PSID households continuing to rent Description of Data: Table displaying the difference-in-difference results comparing neighborhood health trajectories for PSID households entering traditional homeownership versus those of PSID households continuing to rent. [file 12889_2024_20982_MOESM3_ESM.docx]

**Additional File 3. Results of difference-in-differences analysis of PSID households entering traditional homeownership versus PSID households continuing to rent**

|  | **Walkability Score^a^** | **Food Access^b^** | **SVI-SES^c^** | **Life Expectancy^d^** |
| --- | --- | --- | --- | --- |
| Intercept  (SD)  *p-value* | 11.2  (0.16)  *<0.001* | 0.78  (0.02)  *<0.001* | 0.52  (0.01)  *<0.001* | 78.32  (0.18)  *<0.001* |
| Rent-Own  (SD)  *p-value* | -0.61  (0.283)  *0.03* | 0.05  (0.03)  *0.13* | –0.03  (0.02)  *0.22* | 0.29  (0.30)  *0.33* |
| Period  (SD)  *p-value* | 0.03  (0.22)  *0.91* | 0.03  (0.03)  *0.18* | -0.01  (0.02)  *0.74* | 0.10  (0.24)  *0.67* |
| SEH * Period  (SD)  *p-value* | -0.98  (0.42)  *0.02* | -0.12  (0.05)  *0.009* | 0.00  (0.03)  *0.97* | –0.14  (0.40)  *0.73* |
| Treatment Observations 927  Control Observations 1726 | | | | |
| SD, standard deviation  ^a^ Data from Environment Protection Agency National Walkability Index. Ranked quantiles from 1 to 20 (1 = lowest 5%).  ^b^ Data from U.S. Department of Agriculture Food Access Resource Atlas. Binary variable (1 = adequate access to food).  ^c^ Data from Center for Disease Control Social Vulnerability Index, Socioeconomic domain. Percentile from 0 to 1 (1 = highest vulnerability).  ^d^ Data from Center for Disease Control’s U.S. Small-Area Life Expectancy Estimates Project. Life expectancy at birth in years. | | | | |
