# Supplementary material for: Pursuing healthy homeownership: an evaluation of the neighborhood health trajectories of shared equity homeowners
Source: BMC Public Health. 2025 Jan 2;25:11. doi: 10.1186/s12889-024-20982-z (PMC11697963; doi:10.1186/s12889-024-20982-z)
Supplement: Supplementary file 4 — Additional File 4 Assessment of variable stability over time. Description of Data: Table displaying the intraclass correlation (ICC) between various waves of data for the study variables for which multiple years of data were available (food access and the socioeconomic domain of the social vulnerability index). [file 12889_2024_20982_MOESM4_ESM.docx]

**Additional File 4. Assessment of Variable Stability over Time**

| **Variable** | **Comparison Years^a^** | **Intraclass Correlation** |
| --- | --- | --- |
| Food Access^a^ (within 0.5 mile for urban, 10 miles for rural) | 2010, 2015, 2019 | 0.89 |
| Social Vulnerability Index^b^ – Socioeconomic Domain (SVI-SES) | 2010, 2014, 2016, 2018 | 0.92 |

^a^ Year refers to the final year data was collected on the measure. In the case of SVI-SES, the measure is based on the American Community Survey (ACS), with data reflecting a five-year interval ending with the listed year (e.g. 2016 SVI-SES data is based on ACS data from 2012-2016).
